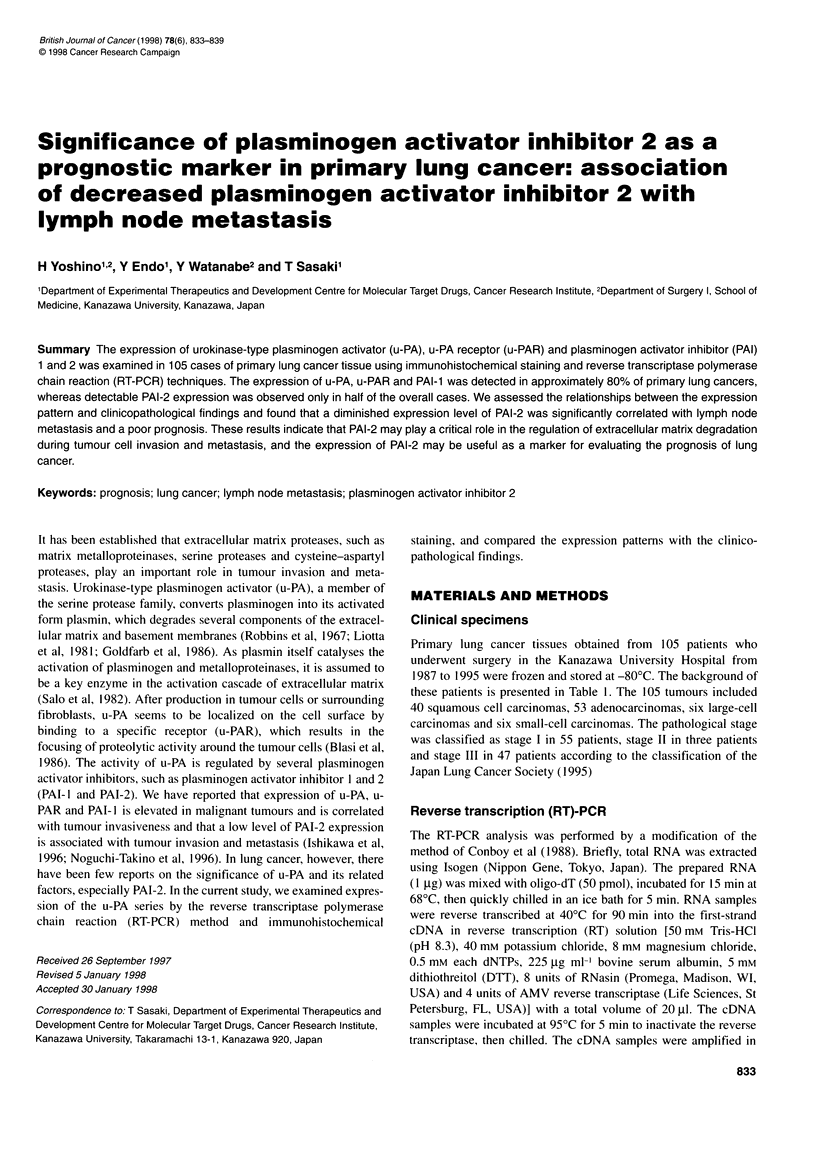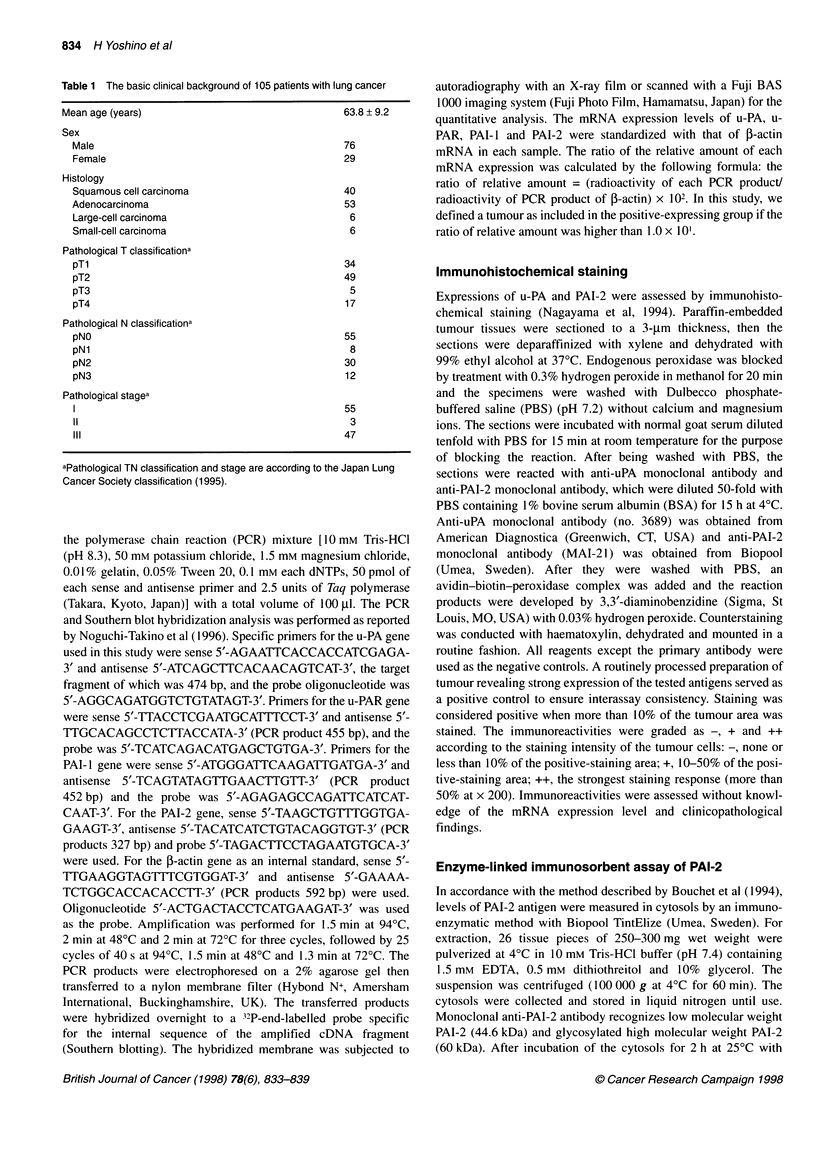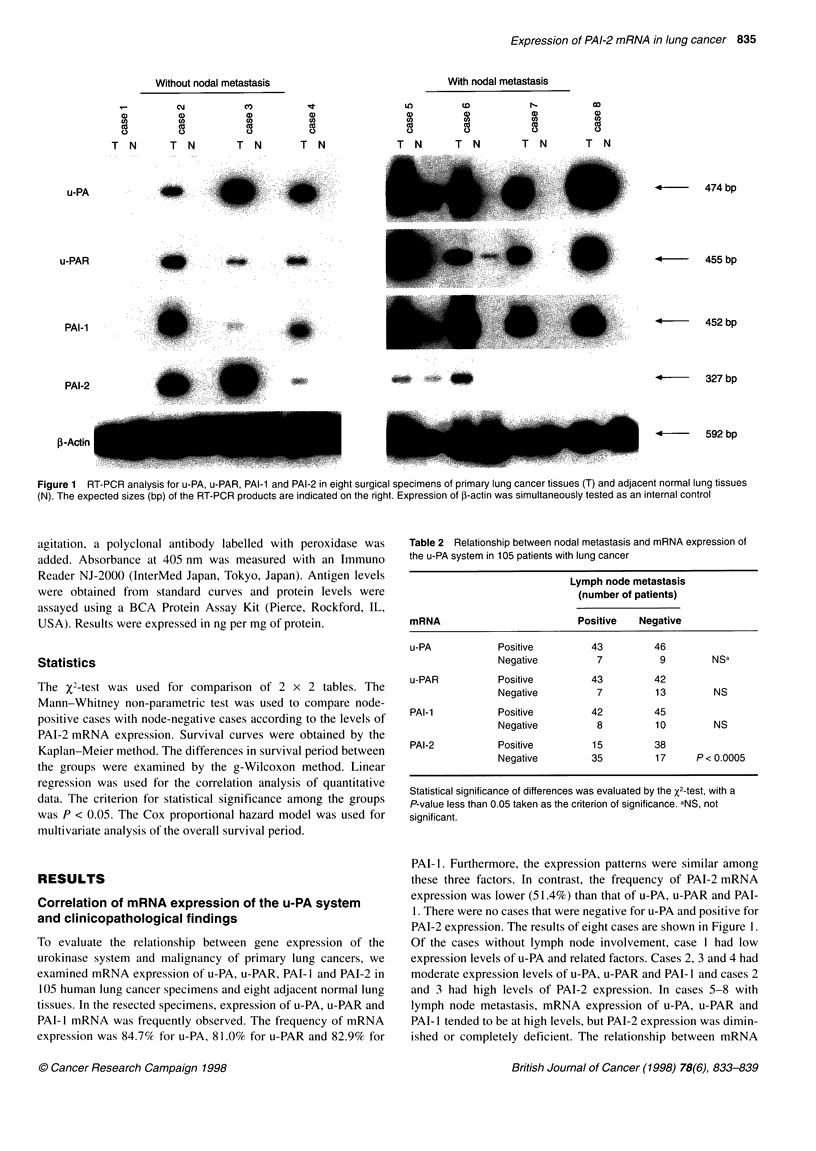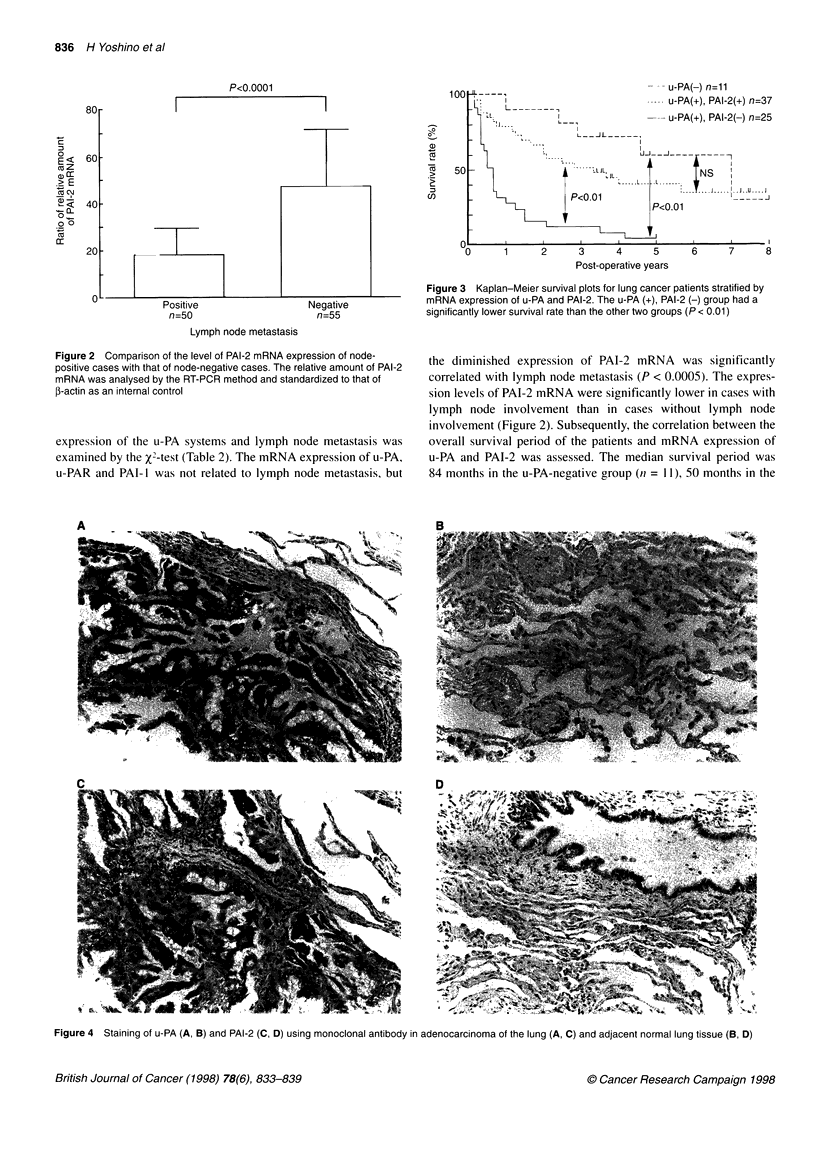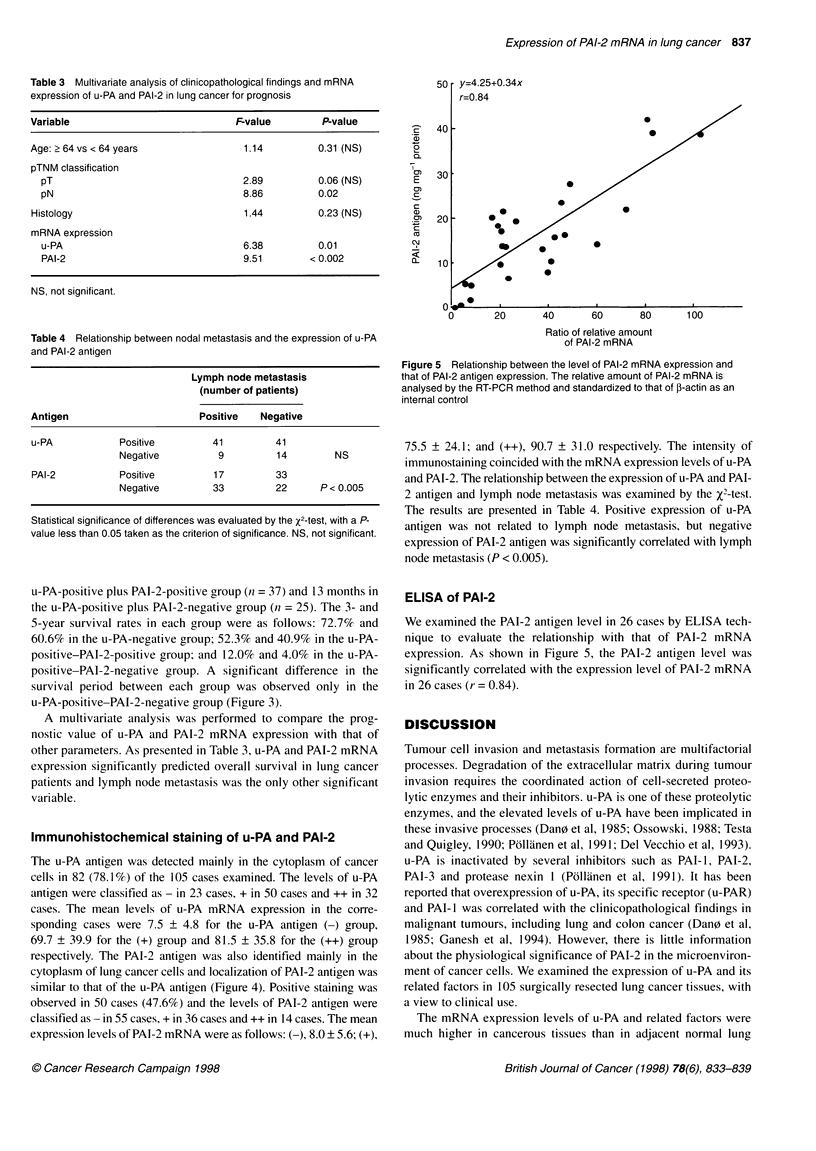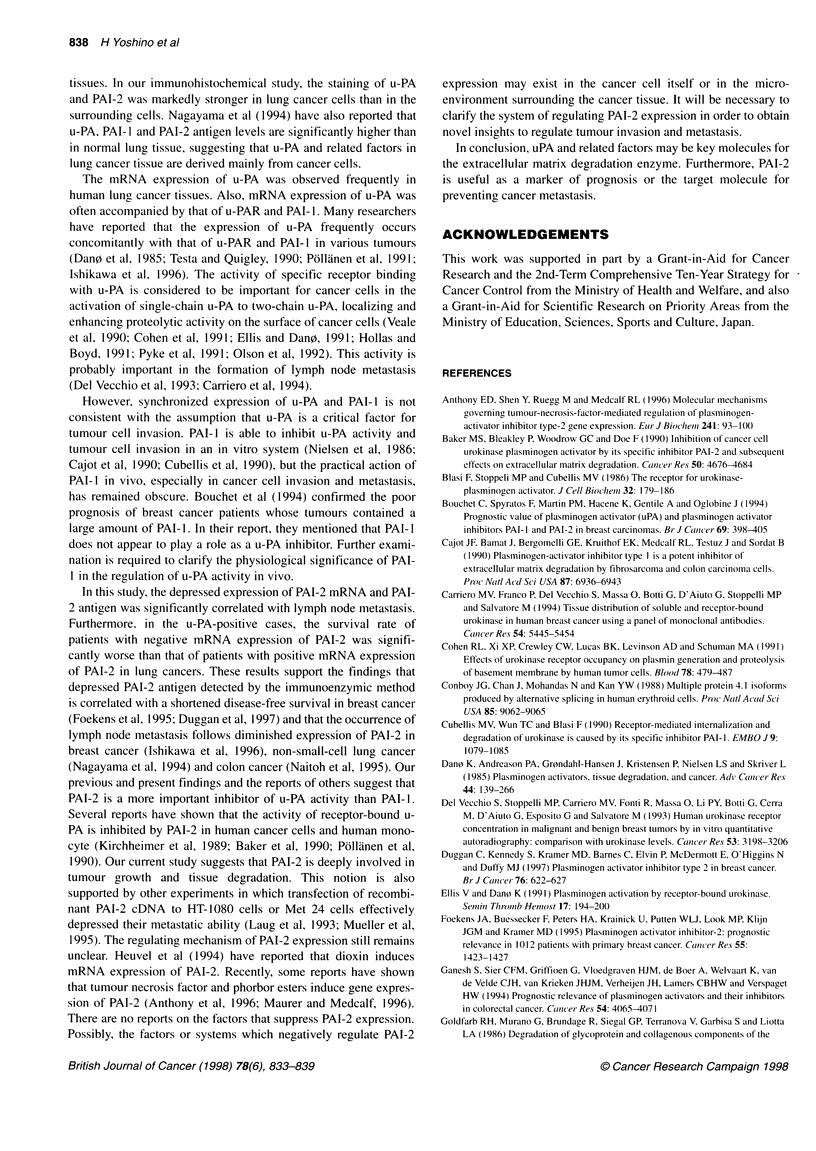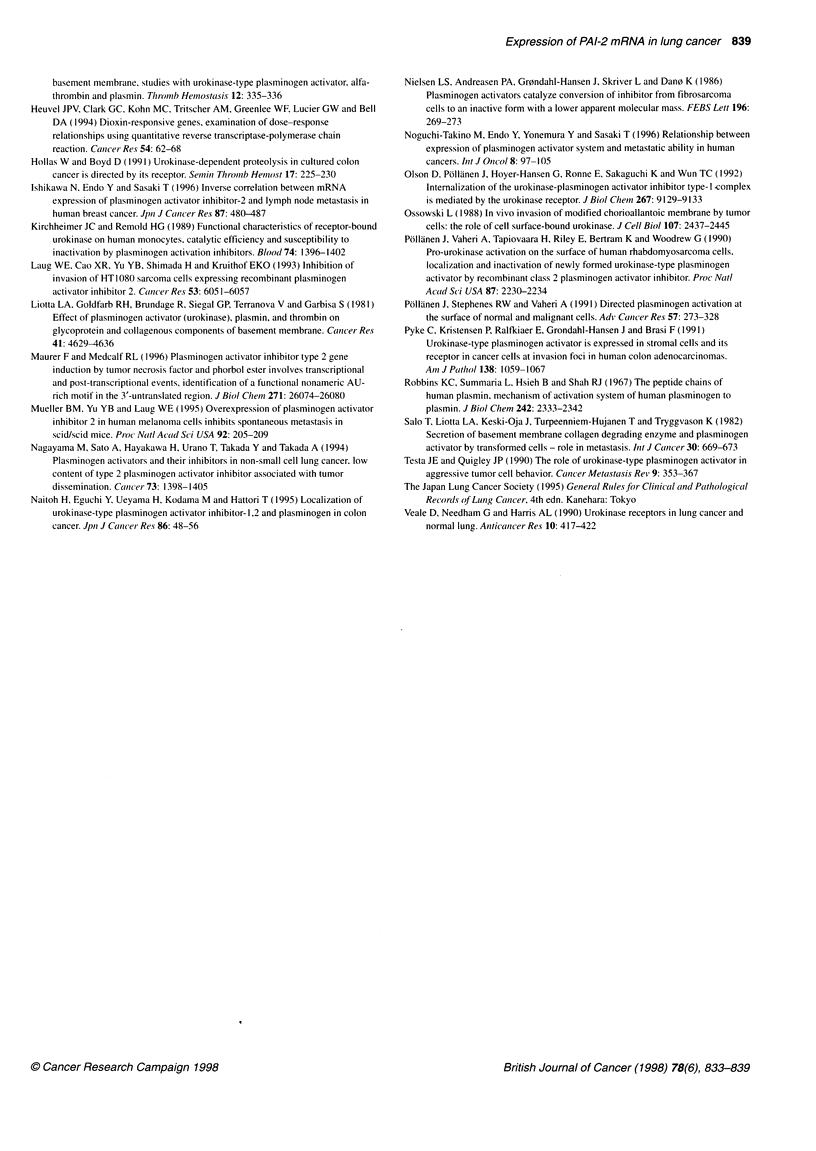# Significance of plasminogen activator inhibitor 2 as a prognostic marker in primary lung cancer: association of decreased plasminogen activator inhibitor 2 with lymph node metastasis.

**DOI:** 10.1038/bjc.1998.588

**Published:** 1998-09

**Authors:** H. Yoshino, Y. Endo, Y. Watanabe, T. Sasaki

**Affiliations:** Department of Experimental Therapeutics and Development Centre for Molecular Target Drugs, Cancer Research Institute, Kanazawa University, Japan.

## Abstract

**Images:**


British Journal of Cancer (1998) 78(6), 833–839
© 1998 Cancer Research Campaign

### Significance of plasminogen activator inhibitor 2 as a prognostic marker in primary lung cancer: association of decreased plasminogen activator inhibitor 2 with lymph node metastasis

**H Yoshino[1,2], Y Endo[1], Y Watanabe[2] and T Sasaki[1]**

[1]Department of Experimental Therapeutics and Development Centre for Molecular Target Drugs, Cancer Research Institute, [2]Department of Surgery I, School of Medicine, Kanazawa University, Kanazawa, Japan

**Summary** The expression of urokinase-type plasminogen activator (u-PA), u-PA receptor (u-PAR) and plasminogen activator inhibitor (PAI) 1 and 2 was examined in 105 cases of primary lung cancer tissue using immunohistochemical staining and reverse transcriptase polymerase chain reaction (RT-PCR) techniques. The expression of u-PA, u-PAR and PAI-1 was detected in approximately 80% of primary lung cancers, whereas detectable PAI-2 expression was observed only in half of the overall cases. We assessed the relationships between the expression pattern and clinicopathological findings and found that a diminished expression level of PAI-2 was significantly correlated with lymph node metastasis and a poor prognosis. These results indicate that PAI-2 may play a critical role in the regulation of extracellular matrix degradation during tumour cell invasion and metastasis, and the expression of PAI-2 may be useful as a marker for evaluating the prognosis of lung cancer.

**Keywords:** prognosis; lung cancer; lymph node metastasis; plasminogen activator inhibitor 2

It has been established that extracellular matrix proteases, such as matrix metalloproteinases, serine proteases and cysteine–aspartyl proteases, play an important role in tumour invasion and metastasis. Urokinase-type plasminogen activator (u-PA), a member of the serine protease family, converts plasminogen into its activated form plasmin, which degrades several components of the extracellular matrix and basement membranes (Robbins et al, 1967; Liotta et al, 1981; Goldfarb et al, 1986). As plasmin itself catalyses the activation of plasminogen and metalloproteinases, it is assumed to be a key enzyme in the activation cascade of extracellular matrix (Salo et al, 1982). After production in tumour cells or surrounding fibroblasts, u-PA seems to be localized on the cell surface by binding to a specific receptor (u-PAR), which results in the focusing of proteolytic activity around the tumour cells (Blasi et al, 1986). The activity of u-PA is regulated by several plasminogen activator inhibitors, such as plasminogen activator inhibitor 1 and 2 (PAI-1 and PAI-2). We have reported that expression of u-PA, u-PAR and PAI-1 is elevated in malignant tumours and is correlated with tumour invasiveness and that a low level of PAI-2 expression is associated with tumour invasion and metastasis (Ishikawa et al, 1996; Noguchi-Takino et al, 1996). In lung cancer, however, there have been few reports on the significance of u-PA and its related factors, especially PAI-2. In the current study, we examined expression of the u-PA series by the reverse transcriptase polymerase chain reaction (RT-PCR) method and immunohistochemical staining, and compared the expression patterns with the clinicopathological findings.

*Received 26 September 1997*
*Revised 5 January 1998*
*Accepted 30 January 1998*

*Correspondence to:* T Sasaki, Department of Experimental Therapeutics and Development Centre for Molecular Target Drugs, Cancer Research Institute, Kanazawa University, Takaramachi 13-1, Kanazawa 920, Japan

### MATERIALS AND METHODS

### Clinical specimens

Primary lung cancer tissues obtained from 105 patients who underwent surgery in the Kanazawa University Hospital from 1987 to 1995 were frozen and stored at –80°C. The background of these patients is presented in Table 1. The 105 tumours included 40 squamous cell carcinomas, 53 adenocarcinomas, six large-cell carcinomas and six small-cell carcinomas. The pathological stage was classified as stage I in 55 patients, stage II in three patients and stage III in 47 patients according to the classification of the Japan Lung Cancer Society (1995)

### Reverse transcription (RT)-PCR

The RT-PCR analysis was performed by a modification of the method of Conboy et al (1988). Briefly, total RNA was extracted using Isogen (Nippon Gene, Tokyo, Japan). The prepared RNA (1 μg) was mixed with oligo-dT (50 pmol), incubated for 15 min at 68°C, then quickly chilled in an ice bath for 5 min. RNA samples were reverse transcribed at 40°C for 90 min into the first-strand cDNA in reverse transcription (RT) solution [50 mM Tris-HCl (pH 8.3), 40 mM potassium chloride, 8 mM magnesium chloride, 0.5 mM each dNTPs, 225 μg $ml^{-1}$ bovine serum albumin, 5 mM dithiothreitol (DTT), 8 units of RNasin (Promega, Madison, WI, USA) and 4 units of AMV reverse transcriptase (Life Sciences, St Petersburg, FL, USA)] with a total volume of 20 μl. The cDNA samples were incubated at 95°C for 5 min to inactivate the reverse transcriptase, then chilled. The cDNA samples were amplified in

**Table 1** The basic clinical background of 105 patients with lung cancer

| | |
|---|---|
| Mean age (years) | 63.8 ± 9.2 |
| Sex | |
| Male | 76 |
| Female | 29 |
| Histology | |
| Squamous cell carcinoma | 40 |
| Adenocarcinoma | 53 |
| Large-cell carcinoma | 6 |
| Small-cell carcinoma | 6 |
| Pathological T classification[a] | |
| pT1 | 34 |
| pT2 | 49 |
| pT3 | 5 |
| pT4 | 17 |
| Pathological N classification[a] | |
| pN0 | 55 |
| pN1 | 8 |
| pN2 | 30 |
| pN3 | 12 |
| Pathological stage[a] | |
| I | 55 |
| II | 3 |
| III | 47 |

[a]Pathological TN classification and stage are according to the Japan Lung Cancer Society classification (1995).

the polymerase chain reaction (PCR) mixture [10 mM Tris-HCl (pH 8.3), 50 mM potassium chloride, 1.5 mM magnesium chloride, 0.01% gelatin, 0.05% Tween 20, 0.1 mM each dNTPs, 50 pmol of each sense and antisense primer and 2.5 units of *Taq* polymerase (Takara, Kyoto, Japan)] with a total volume of 100 μl. The PCR and Southern blot hybridization analysis was performed as reported by Noguchi-Takino et al (1996). Specific primers for the u-PA gene used in this study were sense 5′-AGAATTCACCACCATCGAGA-3′ and antisense 5′-ATCAGCTTCACAACAGTCAT-3′, the target fragment of which was 474 bp, and the probe oligonucleotide was 5′-AGGCAGATGGTCTGTATAGT-3′. Primers for the u-PAR gene were sense 5′-TTACCTCGAATGCATTTCCT-3′ and antisense 5′-TTGCACAGCCTCTTACCATA-3′ (PCR product 455 bp), and the probe was 5′-TCATCAGACATGAGCTGTGA-3′. Primers for the PAI-1 gene were sense 5′-ATGGGATTCAAGATTGATGA-3′ and antisense 5′-TCAGTATAGTTGAACTTGTT-3′ (PCR product 452 bp) and the probe was 5′-AGAGAGCCAGATTCATCAT-CAAT-3′. For the PAI-2 gene, sense 5′-TAAGCTGTTTGGTGA-GAAGT-3′, antisense 5′-TACATCATCTGTACAGGTGT-3′ (PCR products 327 bp) and probe 5′-TAGACTTCCTAGAATGTGCA-3′ were used. For the β-actin gene as an internal standard, sense 5′-TTGAAGGTAGTTTCGTGGAT-3′ and antisense 5′-GAAAA-TCTGGCACCACACCTT-3′ (PCR products 592 bp) were used. Oligonucleotide 5′-ACTGACTACCTCATGAAGAT-3′ was used as the probe. Amplification was performed for 1.5 min at 94°C, 2 min at 48°C and 2 min at 72°C for three cycles, followed by 25 cycles of 40 s at 94°C, 1.5 min at 48°C and 1.3 min at 72°C. The PCR products were electrophoresed on a 2% agarose gel then transferred to a nylon membrane filter (Hybond N+, Amersham International, Buckinghamshire, UK). The transferred products were hybridized overnight to a $^{32}P$-end-labelled probe specific for the internal sequence of the amplified cDNA fragment (Southern blotting). The hybridized membrane was subjected to autoradiography with an X-ray film or scanned with a Fuji BAS 1000 imaging system (Fuji Photo Film, Hamamatsu, Japan) for the quantitative analysis. The mRNA expression levels of u-PA, u-PAR, PAI-1 and PAI-2 were standardized with that of β-actin mRNA in each sample. The ratio of the relative amount of each mRNA expression was calculated by the following formula: the ratio of relative amount = (radioactivity of each PCR product/ radioactivity of PCR product of β-actin) $\times 10^2$. In this study, we defined a tumour as included in the positive-expressing group if the ratio of relative amount was higher than $1.0 \times 10^1$.

### Immunohistochemical staining

Expressions of u-PA and PAI-2 were assessed by immunohistochemical staining (Nagayama et al, 1994). Paraffin-embedded tumour tissues were sectioned to a 3-μm thickness, then the sections were deparaffinized with xylene and dehydrated with 99% ethyl alcohol at 37°C. Endogenous peroxidase was blocked by treatment with 0.3% hydrogen peroxide in methanol for 20 min and the specimens were washed with Dulbecco phosphate-buffered saline (PBS) (pH 7.2) without calcium and magnesium ions. The sections were incubated with normal goat serum diluted tenfold with PBS for 15 min at room temperature for the purpose of blocking the reaction. After being washed with PBS, the sections were reacted with anti-uPA monoclonal antibody and anti-PAI-2 monoclonal antibody, which were diluted 50-fold with PBS containing 1% bovine serum albumin (BSA) for 15 h at 4°C. Anti-uPA monoclonal antibody (no. 3689) was obtained from American Diagnostica (Greenwich, CT, USA) and anti-PAI-2 monoclonal antibody (MAI-21) was obtained from Biopool (Umea, Sweden). After they were washed with PBS, an avidin–biotin–peroxidase complex was added and the reaction products were developed by 3,3′-diaminobenzidine (Sigma, St Louis, MO, USA) with 0.03% hydrogen peroxide. Counterstaining was conducted with haematoxylin, dehydrated and mounted in a routine fashion. All reagents except the primary antibody were used as the negative controls. A routinely processed preparation of tumour revealing strong expression of the tested antigens served as a positive control to ensure interassay consistency. Staining was considered positive when more than 10% of the tumour area was stained. The immunoreactivities were graded as –, + and ++ according to the staining intensity of the tumour cells: –, none or less than 10% of the positive-staining area; +, 10–50% of the positive-staining area; ++, the strongest staining response (more than 50% at × 200). Immunoreactivities were assessed without knowledge of the mRNA expression level and clinicopathological findings.

### Enzyme-linked immunosorbent assay of PAI-2

In accordance with the method described by Bouchet et al (1994), levels of PAI-2 antigen were measured in cytosols by an immunoenzymatic method with Biopool TintElize (Umea, Sweden). For extraction, 26 tissue pieces of 250–300 mg wet weight were pulverized at 4°C in 10 mM Tris-HCl buffer (pH 7.4) containing 1.5 mM EDTA, 0.5 mM dithiothreitol and 10% glycerol. The suspension was centrifuged (100 000 *g* at 4°C for 60 min). The cytosols were collected and stored in liquid nitrogen until use. Monoclonal anti-PAI-2 antibody recognizes low molecular weight PAI-2 (44.6 kDa) and glycosylated high molecular weight PAI-2 (60 kDa). After incubation of the cytosols for 2 h at 25°C with

© Cancer Research Campaign 1998

**Figure 1** RT-PCR analysis for u-PA, u-PAR, PAI-1 and PAI-2 in eight surgical specimens of primary lung cancer tissues (T) and adjacent normal lung tissues (N). The expected sizes (bp) of the RT-PCR products are indicated on the right. Expression of β-actin was simultaneously tested as an internal control agitation, a polyclonal antibody labelled with peroxidase was added. Absorbance at 405 nm was measured with an Immuno Reader NJ-2000 (InterMed Japan, Tokyo, Japan). Antigen levels were obtained from standard curves and protein levels were assayed using a BCA Protein Assay Kit (Pierce, Rockford, IL, USA). Results were expressed in ng per mg of protein.

### Statistics

The $\chi^2$-test was used for comparison of 2 × 2 tables. The Mann–Whitney non-parametric test was used to compare node-positive cases with node-negative cases according to the levels of PAI-2 mRNA expression. Survival curves were obtained by the Kaplan–Meier method. The differences in survival period between the groups were examined by the g-Wilcoxon method. Linear regression was used for the correlation analysis of quantitative data. The criterion for statistical significance among the groups was $P < 0.05$. The Cox proportional hazard model was used for multivariate analysis of the overall survival period.

**Table 2** Relationship between nodal metastasis and mRNA expression of the u-PA system in 105 patients with lung cancer

| mRNA | | Lymph node metastasis (number of patients) Positive | Negative | |
|---|---|---|---|---|
| u-PA | Positive | 43 | 46 | |
| | Negative | 7 | 9 | NS[a] |
| u-PAR | Positive | 43 | 42 | |
| | Negative | 7 | 13 | NS |
| PAI-1 | Positive | 42 | 45 | |
| | Negative | 8 | 10 | NS |
| PAI-2 | Positive | 15 | 38 | |
| | Negative | 35 | 17 | $P < 0.0005$ |

Statistical significance of differences was evaluated by the $\chi^2$-test, with a $P$-value less than 0.05 taken as the criterion of significance. [a]NS, not significant.

### RESULTS

### Correlation of mRNA expression of the u-PA system and clinicopathological findings

To evaluate the relationship between gene expression of the urokinase system and malignancy of primary lung cancers, we examined mRNA expression of u-PA, u-PAR, PAI-1 and PAI-2 in 105 human lung cancer specimens and eight adjacent normal lung tissues. In the resected specimens, expression of u-PA, u-PAR and PAI-1 mRNA was frequently observed. The frequency of mRNA expression was 84.7% for u-PA, 81.0% for u-PAR and 82.9% for PAI-1. Furthermore, the expression patterns were similar among these three factors. In contrast, the frequency of PAI-2 mRNA expression was lower (51.4%) than that of u-PA, u-PAR and PAI-1. There were no cases that were negative for u-PA and positive for PAI-2 expression. The results of eight cases are shown in Figure 1. Of the cases without lymph node involvement, case 1 had low expression levels of u-PA and related factors. Cases 2, 3 and 4 had moderate expression levels of u-PA, u-PAR and PAI-1 and cases 2 and 3 had high levels of PAI-2 expression. In cases 5–8 with lymph node metastasis, mRNA expression of u-PA, u-PAR and PAI-1 tended to be at high levels, but PAI-2 expression was diminished or completely deficient. The relationship between mRNA

© Cancer Research Campaign 1998

**Figure 2** Comparison of the level of PAI-2 mRNA expression of node-positive cases with that of node-negative cases. The relative amount of PAI-2 mRNA was analysed by the RT-PCR method and standardized to that of β-actin as an internal control

**Figure 3** Kaplan–Meier survival plots for lung cancer patients stratified by mRNA expression of u-PA and PAI-2. The u-PA (+), PAI-2 (–) group had a significantly lower survival rate than the other two groups ($P < 0.01$)

expression of the u-PA systems and lymph node metastasis was examined by the $\chi^2$-test (Table 2). The mRNA expression of u-PA, u-PAR and PAI-1 was not related to lymph node metastasis, but the diminished expression of PAI-2 mRNA was significantly correlated with lymph node metastasis ($P < 0.0005$). The expression levels of PAI-2 mRNA were significantly lower in cases with lymph node involvement than in cases without lymph node involvement (Figure 2). Subsequently, the correlation between the overall survival period of the patients and mRNA expression of u-PA and PAI-2 was assessed. The median survival period was 84 months in the u-PA-negative group ($n$ = 11), 50 months in the

**Figure 4** Staining of u-PA (**A**, **B**) and PAI-2 (**C**, **D**) using monoclonal antibody in adenocarcinoma of the lung (**A**, **C**) and adjacent normal lung tissue (**B**, **D**)

 © Cancer Research Campaign 1998

**Table 3** Multivariate analysis of clinicopathological findings and mRNA expression of u-PA and PAI-2 in lung cancer for prognosis

| Variable | *F*-value | *P*-value |
|---|---|---|
| Age: ≥ 64 vs < 64 years | 1.14 | 0.31 (NS) |
| pTNM classification | | |
| pT | 2.89 | 0.06 (NS) |
| pN | 8.86 | 0.02 |
| Histology | 1.44 | 0.23 (NS) |
| mRNA expression | | |
| u-PA | 6.38 | 0.01 |
| PAI-2 | 9.51 | < 0.002 |

NS, not significant.

**Table 4** Relationship between nodal metastasis and the expression of u-PA and PAI-2 antigen

| | | Lymph node metastasis (number of patients) | | |
|---|---|---|---|---|
| Antigen | | Positive | Negative | |
| u-PA | Positive | 41 | 41 | |
| | Negative | 9 | 14 | NS |
| PAI-2 | Positive | 17 | 33 | |
| | Negative | 33 | 22 | *P* < 0.005 |

Statistical significance of differences was evaluated by the $\chi^2$-test, with a *P*-value less than 0.05 taken as the criterion of significance. NS, not significant.

**Figure 5** Relationship between the level of PAI-2 mRNA expression and that of PAI-2 antigen expression. The relative amount of PAI-2 mRNA is analysed by the RT-PCR method and standardized to that of β-actin as an internal control u-PA-positive plus PAI-2-positive group ($n$ = 37) and 13 months in the u-PA-positive plus PAI-2-negative group ($n$ = 25). The 3- and 5-year survival rates in each group were as follows: 72.7% and 60.6% in the u-PA-negative group; 52.3% and 40.9% in the u-PA-positive–PAI-2-positive group; and 12.0% and 4.0% in the u-PA-positive–PAI-2-negative group. A significant difference in the survival period between each group was observed only in the u-PA-positive–PAI-2-negative group (Figure 3).

A multivariate analysis was performed to compare the prognostic value of u-PA and PAI-2 mRNA expression with that of other parameters. As presented in Table 3, u-PA and PAI-2 mRNA expression significantly predicted overall survival in lung cancer patients and lymph node metastasis was the only other significant variable.

### Immunohistochemical staining of u-PA and PAI-2

The u-PA antigen was detected mainly in the cytoplasm of cancer cells in 82 (78.1%) of the 105 cases examined. The levels of u-PA antigen were classified as – in 23 cases, + in 50 cases and ++ in 32 cases. The mean levels of u-PA mRNA expression in the corresponding cases were 7.5 ± 4.8 for the u-PA antigen (–) group, 69.7 ± 39.9 for the (+) group and 81.5 ± 35.8 for the (++) group respectively. The PAI-2 antigen was also identified mainly in the cytoplasm of lung cancer cells and localization of PAI-2 antigen was similar to that of the u-PA antigen (Figure 4). Positive staining was observed in 50 cases (47.6%) and the levels of PAI-2 antigen were classified as – in 55 cases, + in 36 cases and ++ in 14 cases. The mean expression levels of PAI-2 mRNA were as follows: (–), 8.0 ± 5.6; (+), 75.5 ± 24.1; and (++), 90.7 ± 31.0 respectively. The intensity of immunostaining coincided with the mRNA expression levels of u-PA and PAI-2. The relationship between the expression of u-PA and PAI-2 antigen and lymph node metastasis was examined by the $\chi^2$-test. The results are presented in Table 4. Positive expression of u-PA antigen was not related to lymph node metastasis, but negative expression of PAI-2 antigen was significantly correlated with lymph node metastasis ($P$ < 0.005).

### ELISA of PAI-2

We examined the PAI-2 antigen level in 26 cases by ELISA technique to evaluate the relationship with that of PAI-2 mRNA expression. As shown in Figure 5, the PAI-2 antigen level was significantly correlated with the expression level of PAI-2 mRNA in 26 cases ($r$ = 0.84).

### DISCUSSION

Tumour cell invasion and metastasis formation are multifactorial processes. Degradation of the extracellular matrix during tumour invasion requires the coordinated action of cell-secreted proteolytic enzymes and their inhibitors. u-PA is one of these proteolytic enzymes, and the elevated levels of u-PA have been implicated in these invasive processes (Danø et al, 1985; Ossowski, 1988; Testa and Quigley, 1990; Pöllänen et al, 1991; Del Vecchio et al, 1993). u-PA is inactivated by several inhibitors such as PAI-1, PAI-2, PAI-3 and protease nexin 1 (Pöllänen et al, 1991). It has been reported that overexpression of u-PA, its specific receptor (u-PAR) and PAI-1 was correlated with the clinicopathological findings in malignant tumours, including lung and colon cancer (Danø et al, 1985; Ganesh et al, 1994). However, there is little information about the physiological significance of PAI-2 in the microenvironment of cancer cells. We examined the expression of u-PA and its related factors in 105 surgically resected lung cancer tissues, with a view to clinical use.

The mRNA expression levels of u-PA and related factors were much higher in cancerous tissues than in adjacent normal lung

© Cancer Research Campaign 1998

tissues. In our immunohistochemical study, the staining of u-PA and PAI-2 was markedly stronger in lung cancer cells than in the surrounding cells. Nagayama et al (1994) have also reported that u-PA, PAI-1 and PAI-2 antigen levels are significantly higher than in normal lung tissue, suggesting that u-PA and related factors in lung cancer tissue are derived mainly from cancer cells.

The mRNA expression of u-PA was observed frequently in human lung cancer tissues. Also, mRNA expression of u-PA was often accompanied by that of u-PAR and PAI-1. Many researchers have reported that the expression of u-PA frequently occurs concomitantly with that of u-PAR and PAI-1 in various tumours (Danø et al, 1985; Testa and Quigley, 1990; Pöllänen et al, 1991; Ishikawa et al, 1996). The activity of specific receptor binding with u-PA is considered to be important for cancer cells in the activation of single-chain u-PA to two-chain u-PA, localizing and enhancing proteolytic activity on the surface of cancer cells (Veale et al, 1990; Cohen et al, 1991; Ellis and Danø, 1991; Hollas and Boyd, 1991; Pyke et al, 1991; Olson et al, 1992). This activity is probably important in the formation of lymph node metastasis (Del Vecchio et al, 1993; Carriero et al, 1994).

However, synchronized expression of u-PA and PAI-1 is not consistent with the assumption that u-PA is a critical factor for tumour cell invasion. PAI-1 is able to inhibit u-PA activity and tumour cell invasion in an in vitro system (Nielsen et al, 1986; Cajot et al, 1990; Cubellis et al, 1990), but the practical action of PAI-1 in vivo, especially in cancer cell invasion and metastasis, has remained obscure. Bouchet et al (1994) confirmed the poor prognosis of breast cancer patients whose tumours contained a large amount of PAI-1. In their report, they mentioned that PAI-1 does not appear to play a role as a u-PA inhibitor. Further examination is required to clarify the physiological significance of PAI-1 in the regulation of u-PA activity in vivo.

In this study, the depressed expression of PAI-2 mRNA and PAI-2 antigen was significantly correlated with lymph node metastasis. Furthermore, in the u-PA-positive cases, the survival rate of patients with negative mRNA expression of PAI-2 was significantly worse than that of patients with positive mRNA expression of PAI-2 in lung cancers. These results support the findings that depressed PAI-2 antigen detected by the immunoenzymic method is correlated with a shortened disease-free survival in breast cancer (Foekens et al, 1995; Duggan et al, 1997) and that the occurrence of lymph node metastasis follows diminished expression of PAI-2 in breast cancer (Ishikawa et al, 1996), non-small-cell lung cancer (Nagayama et al, 1994) and colon cancer (Naitoh et al, 1995). Our previous and present findings and the reports of others suggest that PAI-2 is a more important inhibitor of u-PA activity than PAI-1. Several reports have shown that the activity of receptor-bound u-PA is inhibited by PAI-2 in human cancer cells and human monocyte (Kirchheimer et al, 1989; Baker et al, 1990; Pöllänen et al, 1990). Our current study suggests that PAI-2 is deeply involved in tumour growth and tissue degradation. This notion is also supported by other experiments in which transfection of recombinant PAI-2 cDNA to HT-1080 cells or Met 24 cells effectively depressed their metastatic ability (Laug et al, 1993; Mueller et al, 1995). The regulating mechanism of PAI-2 expression still remains unclear. Heuvel et al (1994) have reported that dioxin induces mRNA expression of PAI-2. Recently, some reports have shown that tumour necrosis factor and phorbor esters induce gene expression of PAI-2 (Anthony et al, 1996; Maurer and Medcalf, 1996). There are no reports on the factors that suppress PAI-2 expression. Possibly, the factors or systems which negatively regulate PAI-2 expression may exist in the cancer cell itself or in the micro-environment surrounding the cancer tissue. It will be necessary to clarify the system of regulating PAI-2 expression in order to obtain novel insights to regulate tumour invasion and metastasis.

In conclusion, uPA and related factors may be key molecules for the extracellular matrix degradation enzyme. Furthermore, PAI-2 is useful as a marker of prognosis or the target molecule for preventing cancer metastasis.

### ACKNOWLEDGEMENTS

This work was supported in part by a Grant-in-Aid for Cancer Research and the 2nd-Term Comprehensive Ten-Year Strategy for Cancer Control from the Ministry of Health and Welfare, and also a Grant-in-Aid for Scientific Research on Priority Areas from the Ministry of Education, Sciences, Sports and Culture, Japan.

### REFERENCES

Anthony ED, Shen Y, Ruegg M and Medcalf RL (1996) Molecular mechanisms governing tumour-necrosis-factor-mediated regulation of plasminogen-activator inhibitor type-2 gene expression. *Eur J Biochem* **241**: 93–100

Baker MS, Bleakley P, Woodrow GC and Doe F (1990) Inhibition of cancer cell urokinase plasminogen activator by its specific inhibitor PAI-2 and subsequent effects on extracellular matrix degradation. *Cancer Res* **50**: 4676–4684

Blasi F, Stoppeli MP and Cubellis MV (1986) The receptor for urokinase-plasminogen activator. *J Cell Biochem* **32**: 179–186

Bouchet C, Spyratos F, Martin PM, Hacene K, Gentile A and Oglobine J (1994) Prognostic value of plasminogen activator (uPA) and plasminogen activator inhibitors PAI-1 and PAI-2 in breast carcinomas. *Br J Cancer* **69**: 398–405

Cajot JF, Bamat J, Bergomelli GE, Kruithof EK, Medcalf RL, Testuz J and Sordat B (1990) Plasminogen-activator inhibitor type 1 is a potent inhibitor of extracellular matrix degradation by fibrosarcoma and colon carcinoma cells. *Proc Natl Acd Sci USA* **87**: 6936–6943

Carriero MV, Franco P, Del Vecchio S, Massa O, Botti G, D'Aiuto G, Stoppelli MP and Salvatore M (1994) Tissue distribution of soluble and receptor-bound urokinase in human breast cancer using a panel of monoclonal antibodies. *Cancer Res* **54**: 5445–5454

Cohen RL, Xi XP, Crewley CW, Lucas BK, Levinson AD and Schuman MA (1991) Effects of urokinase receptor occupancy on plasmin generation and proteolysis of basement membrane by human tumor cells. *Blood* **78**: 479–487

Conboy JG, Chan J, Mohandas N and Kan YW (1988) Multiple protein 4.1 isoforms produced by alternative splicing in human erythroid cells. *Proc Natl Acad Sci USA* **85**: 9062–9065

Cubellis MV, Wun TC and Blasi F (1990) Receptor-mediated internalization and degradation of urokinase is caused by its specific inhibitor PAI-1. *EMBO J* **9**: 1079–1085

Danø K, Andreason PA, Grøndahl-Hansen J, Kristensen P, Nielsen LS and Skriver L (1985) Plasminogen activators, tissue degradation, and cancer. *Adv Cancer Res* **44**: 139–266

Del Vecchio S, Stoppelli MP, Carriero MV, Fonti R, Massa O, Li PY, Botti G, Cerra M, D'Aiuto G, Esposito G and Salvatore M (1993) Human urokinase receptor concentration in malignant and benign breast tumors by in vitro quantitative autoradiography: comparison with urokinase levels. *Cancer Res* **53**: 3198–3206

Duggan C, Kennedy S, Kramer MD, Barnes C, Elvin P, McDermott E, O'Higgins N and Duffy MJ (1997) Plasminogen activator inhibitor type 2 in breast cancer. *Br J Cancer* **76**: 622–627

Ellis V and Danø K (1991) Plasminogen activation by receptor-bound urokinase. *Semin Thromb Hemost* **17**: 194–200

Foekens JA, Buessecker F, Peters HA, Krainick U, Putten WLJ, Look MP, Klijn JGM and Kramer MD (1995) Plasminogen activator inhibitor-2: prognostic relevance in 1012 patients with primary breast cancer. *Cancer Res* **55**: 1423–1427

Ganesh S, Sier CFM, Griffioen G, Vloedgraven HJM, de Boer A, Welvaart K, van de Velde CJH, van Krieken JHJM, Verheijen JH, Lamers CBHW and Verspaget HW (1994) Prognostic relevance of plasminogen activators and their inhibitors in colorectal cancer. *Cancer Res* **54**: 4065–4071

Goldfarb RH, Murano G, Brundage R, Siegal GP, Terranova V, Garbisa S and Liotta LA (1986) Degradation of glycoprotein and collagenous components of the

© Cancer Research Campaign 1998

basement membrane, studies with urokinase-type plasminogen activator, alfa-thrombin and plasmin. *Thromb Hemostasis* **12**: 335–336

Heuvel JPV, Clark GC, Kohn MC, Tritscher AM, Greenlee WF, Lucier GW and Bell DA (1994) Dioxin-responsive genes, examination of dose–response relationships using quantitative reverse transcriptase-polymerase chain reaction. *Cancer Res* **54**: 62–68

Hollas W and Boyd D (1991) Urokinase-dependent proteolysis in cultured colon cancer is directed by its receptor. *Semin Thromb Hemost* **17**: 225–230

Ishikawa N, Endo Y and Sasaki T (1996) Inverse correlation between mRNA expression of plasminogen activator inhibitor-2 and lymph node metastasis in human breast cancer. *Jpn J Cancer Res* **87**: 480–487

Kirchheimer JC and Remold HG (1989) Functional characteristics of receptor-bound urokinase on human monocytes, catalytic efficiency and susceptibility to inactivation by plasminogen activation inhibitors. *Blood* **74**: 1396–1402

Laug WE, Cao XR, Yu YB, Shimada H and Kruithof EKO (1993) Inhibition of invasion of HT1080 sarcoma cells expressing recombinant plasminogen activator inhibitor 2. *Cancer Res* **53**: 6051–6057

Liotta LA, Goldfarb RH, Brundage R, Siegal GP, Terranova V and Garbisa S (1981) Effect of plasminogen activator (urokinase), plasmin, and thrombin on glycoprotein and collagenous components of basement membrane. *Cancer Res* **41**: 4629–4636

Maurer F and Medcalf RL (1996) Plasminogen activator inhibitor type 2 gene induction by tumor necrosis factor and phorbol ester involves transcriptional and post-transcriptional events, identification of a functional nonameric AU-rich motif in the 3′-untranslated region. *J Biol Chem* **271**: 26074–26080

Mueller BM, Yu YB and Laug WE (1995) Overexpression of plasminogen activator inhibitor 2 in human melanoma cells inhibits spontaneous metastasis in scid/scid mice. *Proc Natl Acad Sci USA* **92**: 205–209

Nagayama M, Sato A, Hayakawa H, Urano T, Takada Y and Takada A (1994) Plasminogen activators and their inhibitors in non-small cell lung cancer, low content of type 2 plasminogen activator inhibitor associated with tumor dissemination. *Cancer* **73**: 1398–1405

Naitoh H, Eguchi Y, Ueyama H, Kodama M and Hattori T (1995) Localization of urokinase-type plasminogen activator inhibitor-1,2 and plasminogen in colon cancer. *Jpn J Cancer Res* **86**: 48–56

Nielsen LS, Andreasen PA, Grøndahl-Hansen J, Skriver L and Danø K (1986) Plasminogen activators catalyze conversion of inhibitor from fibrosarcoma cells to an inactive form with a lower apparent molecular mass. *FEBS Lett* **196**: 269–273

Noguchi-Takino M, Endo Y, Yonemura Y and Sasaki T (1996) Relationship between expression of plasminogen activator system and metastatic ability in human cancers. *Int J Oncol* **8**: 97–105

Olson D, Pöllänen J, Hoyer-Hansen G, Ronne E, Sakaguchi K and Wun TC (1992) Internalization of the urokinase-plasminogen activator inhibitor type-1 complex is mediated by the urokinase receptor. *J Biol Chem* **267**: 9129–9133

Ossowski L (1988) In vivo invasion of modified chorioallantoic membrane by tumor cells: the role of cell surface-bound urokinase. *J Cell Biol* **107**: 2437–2445

Pöllänen J, Vaheri A, Tapiovaara H, Riley E, Bertram K and Woodrew G (1990) Pro-urokinase activation on the surface of human rhabdomyosarcoma cells, localization and inactivation of newly formed urokinase-type plasminogen activator by recombinant class 2 plasminogen activator inhibitor. *Proc Natl Acad Sci USA* **87**: 2230–2234

Pöllänen J, Stephenes RW and Vaheri A (1991) Directed plasminogen activation at the surface of normal and malignant cells. *Adv Cancer Res* **57**: 273–328

Pyke C, Kristensen P, Ralfkiaer E, Grondahl-Hansen J and Brasi F (1991) Urokinase-type plasminogen activator is expressed in stromal cells and its receptor in cancer cells at invasion foci in human colon adenocarcinomas. *Am J Pathol* **138**: 1059–1067

Robbins KC, Summaria L, Hsieh B and Shah RJ (1967) The peptide chains of human plasmin, mechanism of activation system of human plasminogen to plasmin. *J Biol Chem* **242**: 2333–2342

Salo T, Liotta LA, Keski-Oja J, Turpeenniem-Hujanen T and Tryggvason K (1982) Secretion of basement membrane collagen degrading enzyme and plasminogen activator by transformed cells – role in metastasis. *Int J Cancer* **30**: 669–673

Testa JE and Quigley JP (1990) The role of urokinase-type plasminogen activator in aggressive tumor cell behavior. *Cancer Metastasis Rev* **9**: 353–367

The Japan Lung Cancer Society (1995) *General Rules for Clinical and Pathological Records of Lung Cancer*, 4th edn. Kanehara: Tokyo

Veale D, Needham G and Harris AL (1990) Urokinase receptors in lung cancer and normal lung. *Anticancer Res* **10**: 417–422

© Cancer Research Campaign 1998